# Effects of Edible Coating and Modified Atmosphere Technology on the Physiology and Quality of Mangoes after Low-Temperature Transportation at 13 °C in Vibration Mitigation Packaging

**DOI:** 10.3390/plants10112432

**Published:** 2021-11-11

**Authors:** Saichao Wei, Jun Mei, Jing Xie

**Affiliations:** 1 College of Food Science and Technology, Shanghai Ocean University, Shanghai 201306, China; m190300742@st.shou.edu.cn (S.W.); jmei@shou.edu.cn (J.M.); 2 National Experimental Teaching Demonstration Center for Food Science and Engineering, Shanghai Ocean University, Shanghai 201306, China; 3 Shanghai Professional Technology Service Platform on Cold Chain Equipment Performance and Energy Saving Evaluation, Shanghai 201306, China

**Keywords:** *Mangifera indica* L., simulated transport vibration, low-temperature storage, packaging method, edible coating, modified atmosphere, quality

## Abstract

The mango is an important tropical fruit in the world, but it is easily perishable after harvest. In order to investigate the effect of the compound preservation technology on the physiology and quality of mangoes during transportation and storage, mangoes were treated with different packaging and preservation methods. All mangoes were subjected to simulated transportation by a vibration table for 24 h (180 r/min, 13 °C), and stored at 13 °C. The changes in the color, physicochemical characteristics, quality, and antioxidant-related enzymes of the mangoes were measured. The results show that the shelf life of inflatable bag packing (CK) was only 24 d, while the other treatments could be 30 d. The inflatable bag packing with modified atmosphere packaging (MAP) treatment (HPM) had the lowest yellowing degree (12.5%), disease index (34.4%), and mass loss (2.95%), at 30 d. Compared with the CK, the compound treatment containing MAP prolonged the peak respiration of the mangoes by 6 d and suppressed the increase in the total soluble solids and relative conductivity. Meanwhile, the HPM could effectively maintain moisture content, firmness, titratable acid, vitamin C, and the peroxidase and superoxide dismutase content, indicating that the treatment could maintain the better quality and antioxidation ability of mangoes. In summary, the MAP compound treatment better maintained the commercial characteristics of the mangoes, followed by the edible coating compound treatment. The results provide a theoretical reference for mango cushioning packaging and postharvest storage technology.

## 1. Introduction

Mango (*Mangifera indica* L.) is one of the most important tropical fruits in the world because of its economic importance in international trade [1]. Mangoes have a sweet and sour taste, unique flavor, high nutritional value, and are mostly consumed fresh [2,3]. However, mangoes are perishable fruits, and fungal infestation (e.g., *Colletotrichum gloeosporioides Penz.*, and *Botryodiplodia theobromae Pat.*) is one of the main reasons for the loss of commercial characteristics [4,5]. Nutrients, such as the vitamins, titratable acids, and total soluble solids in mangoes, decline rapidly after the peak of respiration [6]. In addition, mangoes need to be transported over long distances before being sold at the terminal. The mechanical damage in this process accelerates the rate of ripening and aging, decreasing the shelf life of mangoes [7,8].

Adequate packaging can reduce the physical damage of the fruit during transportation and ensure a better appearance [9]. At present, mangoes are packaged in polyethylene foam mesh sleeves, and placed in polystyrene foam boxes, except for high-end consumer products. However, according to our previous research, commercial sleeve wrapping has limited protective effect on mangoes. It could not significantly reduce the damage to the fruit, which is not conducive to the postharvest storage of mangoes.

Temperature is one of the most important factors in maintaining the characteristics and quality of mangoes [10]. Low temperatures not only reduce the respiration rate of climacteric fruits, such as mango and dragon fruit, but also inhibit the activity of spoilage bacteria [11]. Thus, low temperatures can effectively extend the storage periods of fruits. However, if the temperature is too low, the fruit will suffer damage and the different symptoms of chilling injury will appear [12]. Most studies find that the temperature threshold for chilling injury in mangoes is below 10–13 °C [13].

Fungicide treatment is another effective way to avoid fungal attacks on mangoes and does not adversely affect their appearance [14], but the abuse of these drugs can lead to the development of resistant populations of the fungi. In addition, there is a growing awareness of the dangers these compounds pose to humans and the environment. The edible coating is proven to be safe and environmentally friendly for the postharvest preservation of fruits [15,16]. The substrates of these coatings contain polysaccharides, proteins, and lipids. Among them, chitosan has been the most studied because of its good film-forming ability and antimicrobial activity [17]. The addition of different bioactive substances (e.g., curcumin, natamycin, etc.), and the improvement of the film-forming performance of the compound edible coatings, are the current research hotspots. The compound edible coating has an obvious preservation effect in the application of pummelo [18], fig fruit [19], apple [20], and other fruits.

Modified atmosphere packaging (MAP) is an effective method for extending the storage periods of fruits. This technology inhibits the growth of microorganisms, delays the rate of postharvest metabolism, and reduces the consumption of nutrients by changing the ratio of gases in the storage environment [21]. Thus, it can extend the storage periods of fruits and maintain their quality. However, earlier studies have shown that there is a significant interaction effect between oxygen, carbon dioxide, temperature, and other factors in the storage environment [22]. Therefore, it is necessary to find suitable gas preservation conditions according to the characteristics of different products.

In our previous studies, we obtained better packaging methods, compound edible coatings, and modified atmosphere packaging conditions [23] individually for mangoes (same as the Materials and Treatment Section description). In this paper, we focused on the protective effect of the above three compound preservation technologies on mangoes during transportation and storage at a low temperature (13 °C), and the changes in the physiology and quality parameters of the mangoes.

## 2. Results

### 2.1. Fruit Color and Disease Index

**Figure 1:**
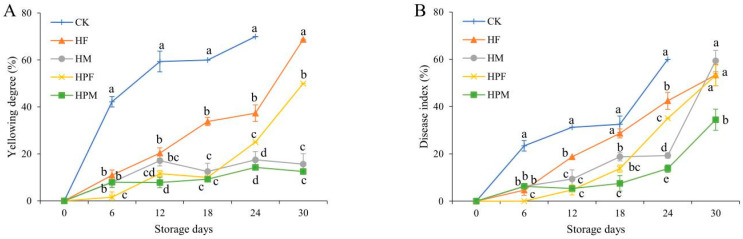
Yellowing degree (**A**), and disease index (**B**), of mangoes with different treatments during 30 days at 13 °C. Treatments: CK (Control): Inflatable bag packaging + Vibration; HF: Coating + Foam mesh sleeve packaging + Vibration; HM: Foam mesh sleeve packaging + Vibration + MAP; HPF: Coating + Inflatable bag packaging + Vibration; and HPM: Inflatable bag packaging + Vibration + MAP. Different letters indicate significant differences between treatments for the same storage time by Duncan’s multiple range tests (*p* < 0.05).

The control of the mangoes did not have edible value after 24 days of cold storage. There was a significant difference in the yellowing degree of the different treatments. The yellowing degree of mango fruits increased significantly (*p* < 0.05) at whole storage (Figure 1A). The lowest yellowing degree was found in the HPM (12.5%), but it did not produce a significant difference from the HM at Days 18 to 30.

The effect of the compound preservation treatments on the disease index of the mangoes was significant (*p* < 0.05). As the storage time increased, the disease index of the mangoes tended to increase (Figure 1B). The highest disease index (60.0%) was observed at the end of storage in the CK. The disease index of the mangoes in the treatment was lower, and the HPM (34.4%) was significantly lower than those of other treatments.

The initial L*, a*, b*, and Δ*E* values (0 day) of the mangoes were 58.9 ± 0.48, −4.65 ± 0.07, 45.2 ± 1.16, and 51.6 ± 1.23, respectively. During low-temperature storage, the different treatments had a significant effect on the L* and b* values of the mangoes (*p* < 0.05), with a decreasing trend. Mangoes treated with the HPM and HM had significantly higher L* values than the CK on the 24th day of storage (Table 1). The highest b* value was found in the pulp of mangoes treated with the HPM. The a* and Δ*E* values gradually increased, with significant differences between the treatments. Mangoes from different treatments showed a lower a* value compared to the CK. The Δ*E* value of the HF and HM were significantly higher and lower than those of other treatments, respectively.

### 2.2. Physicochemical Analysis

**Figure 2:**
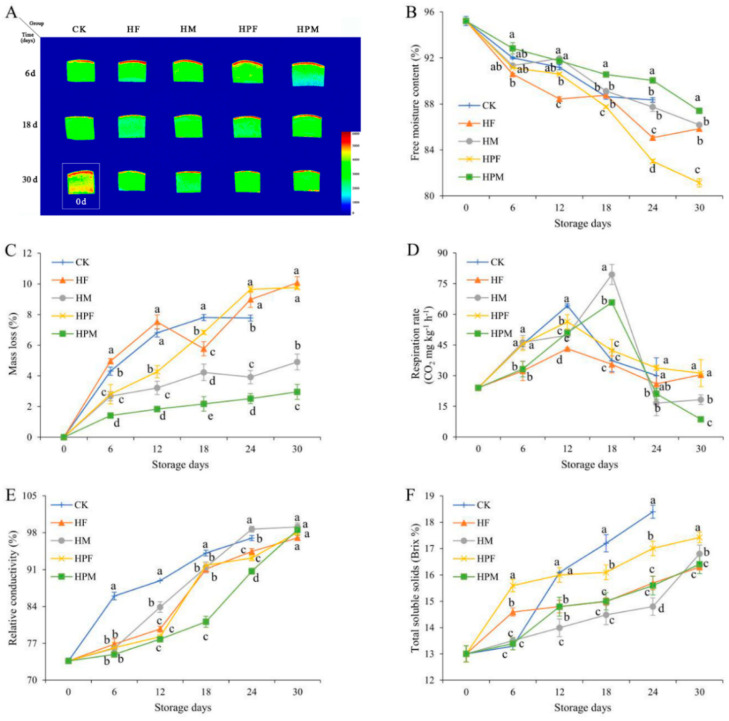
Moisture distribution (**A**), free moisture content (**B**), mass loss (**C**), respiration rate (**D**), relative conductivity (**E**), and total soluble solids (**F**) of mangoes with different treatments during 30 days at 13 °C. Treatments: CK (Control): Inflatable bag packaging + Vibration; HF: Coating + Foam mesh sleeve packaging + Vibration; HM: Foam mesh sleeve packaging + Vibration + MAP; HPF: Coating + Inflatable bag packaging + Vibration; and HPM: Inflatable bag packaging + Vibration + MAP. Different letters indicate significant differences between treatments for the same storage time by Duncan’s multiple range tests (*p* < 0.05).

The variation in the moisture distribution of the mangoes with the storage time is shown in Figure 2A. The moisture content was highest in the peel area, and the pulp moisture was significantly reduced (red or yellow color disappeared) during storage. Different treatments had significant effects on the free moisture distribution of the mangoes, and the HPM retained more peel and pulp moisture at the end of storage.

The compound treatments had a significant effect (*p* < 0.05) on the free moisture content of the mangoes. The free moisture content kept decreasing during the storage, consistent with the trend of moisture distribution shown in Figure 2A. The HPM was optimal, with 87.40% of free moisture at the end of storage, which was significantly higher than that of the HPF (81.15%).

The mass loss of mangoes increased significantly throughout the storage period (*p* < 0.05). The HF had the highest mass loss (10.07%) at whole storage, and the HPF had a higher mass loss than that of the CK on the 24th day. The rate of mass loss was significantly reduced for the HM and HPM, with the HPM showing only 2.95% mass loss at the end of storage (Figure 2C).

The effect of the compound treatments on the respiration rate of the mangoes is shown in Figure 2D. The CK, HF, and HPF reached the peak respiration rate at the 12th day of storage, but the respiration rate of the CK was, significantly, the highest, compared to those of the other treatments. The HM and HPM delayed the peak of mango respiration until Day 18; however, the respiration rate was elevated compared to the CK.

The relative conductivity of the mangoes increased significantly (*p* < 0.05) during the 30-day storage period. The initial mean relative conductivity was 73.6%, all treatments exceeded 97%, and the difference was not significant at the end of storage. However, the different treatments inhibited the increase in the relative conductivity until Day 18 of storage (Figure 2E).

The changes in the total soluble solids of the mangoes during storage are shown in Figure 2F. The total soluble solids of the CK increased rapidly after the 6th day of storage and reached a maximum value of 18.4% on the 24th day. The compound treatments were able to suppress the increase in the total soluble solid content to varying degrees.

### 2.3. Quality Analysis

**Figure 3:**
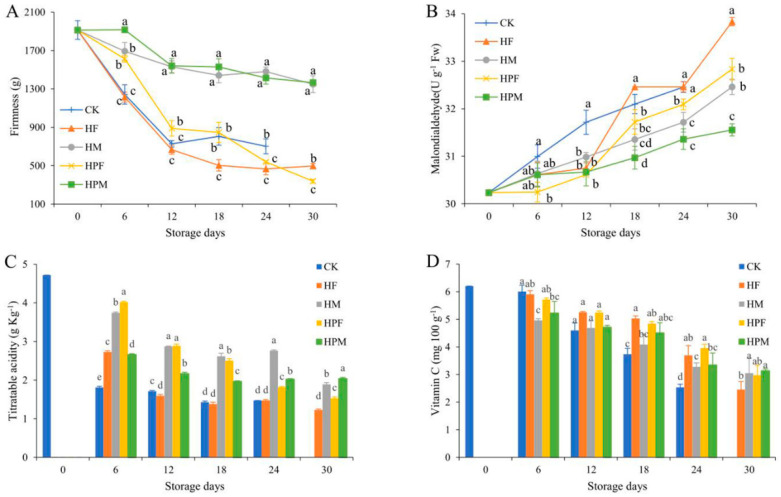
Firmness (**A**), malondialdehyde (**B**), titratable acid (**C**), and vitamin C (**D**) contents of mangoes with different treatments during 30 days at 13 °C. Treatments: CK (Control): Inflatable bag packaging + Vibration; HF: Coating + Foam mesh sleeve packaging + Vibration; HM: Foam mesh sleeve packaging + Vibration + MAP; HPF: Coating + Inflatable bag packaging + Vibration; and HPM: Inflatable bag packaging + Vibration + MAP. Different letters indicate significant differences between treatments for the same storage time by Duncan’s multiple range tests (*p* < 0.05).

The compound treatments had a significant effect on the mangoes’ firmness (*p* < 0.05). The HM and HPM compound treatments significantly maintained the firmness of the mangoes, as seen in Figure 3A. The edible coating compound treatment had less effect on fruit firmness and was even less effective than the CK in preserving firmness after 12 days.

The malondialdehyde (MAD) content of the mangoes increased with the increasing storage time (*p* < 0.05). The treatments did not differ significantly in the effect of MDA inhibition during the prestorage period. The HF and HPF showed a rapid increase in MDA content after the 12th day, with the HF reaching the highest level on the 30th day (33.8 U g^−1^ FW). The MDA content of the HPM was lower than those of the other experimental treatments (Figure 3B).

The variation of the titratable acid (TA) content is shown in Figure 3C. The TA content of the CK decreased rapidly from 0 to 6 days, followed by a smaller decrease. The compound treatments significantly inhibited the decrease of TA, especially the HM and HPF. The TA content of the HPM was significantly higher than those of other treatments on the 30th day (*p* < 0.05).

The vitamin C (VC) content of the mangoes declined continuously during the 30 days of storage. The edible coating compound treatment effectively reduced the loss of VC during low-temperature storage until the 24th day, and the MAP compound treatment was the second most effective (Figure 3D).

### 2.4. Antioxidant-Related Enzyme Analysis

**Figure 4:**
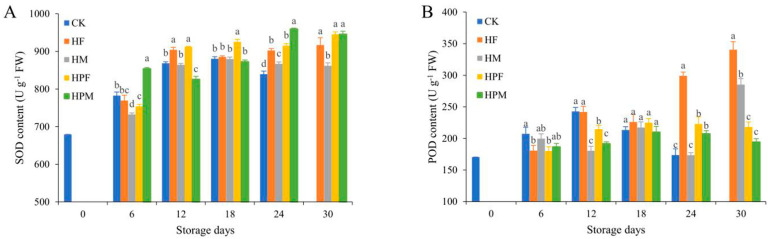
Superoxide dismutase (SOD) (**A**), and peroxidase (POD) (**B**), of mangoes with different treatments during 30 days at 13 °C. Treatments: CK (Control): Inflatable bag packaging + Vibration; HF: Coating + Foam mesh sleeve packaging + Vibration; HM: Foam mesh sleeve packaging + Vibration + MAP; HPF: Coating + Inflatable bag packaging + Vibration; and HPM: Inflatable bag packaging + Vibration + MAP. Different letters indicate significant differences between treatments for the same storage time by Duncan’s multiple range tests (*p* < 0.05).

The initial superoxide dismutase (SOD) content of the mangoes was 679.3 U g^−1^ fresh weight and increased during the experimental storage (Figure 4A). Both the HF and HPF were significantly higher than the CK after Day 12. The HPM was significantly higher than the CK at Days 6 and 24 (*p* < 0.05), and the SOD content of the HM was lower.

The peroxidase (POD) content of the CK increased during the first 12 days and decreased afterward (Figure 4B). The POD content of the treatments was reduced until Day 12, compared to the CK. However, the POD content of the treatments increased after Day 18, especially in the HF, which was significantly higher than those of other treatments at Day 30 of the storage (*p* < 0.05).

## 3. Discussion

The appearance of fresh fruit is one of the most important factors for consumers when purchasing. Mango is a tropical fruit that is usually harvested when their maturity is between 60% and 70% [24]. As the fruit ripens, the color of the mangoes changes accordingly. The yellowing degree of mangoes is due to changes in the pigmentation, which is because of the degradation of the chlorophyll and the synthesis of substances, such as carotenoids [1]. Su et al. reported that, during fruit ripening, chlorophyll was degraded by respiration, light, and other factors. Meanwhile, substances, such as carotene, lutein, and lycopene, were produced from precursor substances [25]. The significant reduction in the yellowing degree of the treatments may indicate that the postripening effect of the mangoes was slowed down throughout the storage period. The physiological and biochemical retardation of the mangoes was caused by the interaction of the protection of the inflatable bag packaging, the edible coating, and the MAP treatments under low-temperature conditions [26,27]. On the basis of the results in Figure 1A, the mangoes treated with edible coating also turned more than 50% yellow at the end of storage. It was found that the MAP compound treatment had a more significant effect on the maintenance of the yellowing degree of the mangoes.

Mangoes are susceptible to fungal attack even before harvesting, which are present in deep or nuclear tissues [28]. As the postripening process accelerates, fungi, such as *Colletotrichum gloeosporioides Penz.* and *Botryodiplodia theobromae Pat.*, can cause disease spots (dark brown spots or mildew) on the surface of mangoes until they rot [29]. Transportation and transfer processes can also damage the tissues and accelerate the fruit decay process [30]. In the compound treatments, the chitosan matrix of the edible coating had some fungal inhibitory effect. The added natamycin was able to bind to the ergosterol moiety of the fungus, leading to cell membrane distortion and death [31]. The results in Figure 2B also indicate that the edible coating compound treatment reduced the occurrence of mango spots. However, the MAP compound treatment showed better results, with an 18.9% reduction in the disease index for the HPM compared to the HF. This may be because the MAP treatment changed the gas ratio in the storage environment and inhibited the growth of the fungi.

The color characteristics of the fruit flesh can allow for an evaluation of the maturity and quality. It is also an important factor influencing consumer preference. Postripening makes mangoes edible after harvest, and this process is accompanied by changes in the fruit color, texture, and flavor. The color of the mango pulp was yellowish, and the brightness of the pulp became darker, which confirmed that the fruit was ripening. This is consistent with the study of Ebrahimi et al. [1]. In addition, the loss of moisture also affected the color characteristics of the fruit [32]. The edible coating compound treatment reduced the fruit respiration rate, but the loss of free moisture and fruit mass was more than those of the MAP compound treatment. The low oxygen conditions adopted by the MAP inhibited the activity of degrading enzymes, such as chlorophyllase, thereby achieving the effect of preserving the green and delaying the ripening process of the fruit [25]. This is another important reason why the HPM compound treatment has a better preservation effect.

Low-field nuclear magnetic resonance (LF-NMR) technology can accurately detect different properties and the contents of the moisture based on the differences in the relaxation times of the hydrogen atoms in a magnetic field [33]. The nuclear magnetic resonance imaging technique can obtain information on the internal moisture distribution and structure of the sample [34]. Transpiration and respiration are important causes of moisture loss in fruits, and the moisture inside the fruit is constantly diffusing into the air from the surface of the peel [35,36]. Figure 2A dynamically shows the continuous migration of moisture from the mango pulp to the peel, a result corroborated by the trend of the mangoes’ free moisture variation (Figure 2B). The HPM significantly inhibited the reduction of the free moisture content in the early storage period, mainly due to the closed environment created by the MAP treatment [37]. The free moisture content of the edible coating compound treatment decreased more during the storage, which could be caused by the adsorption of moisture by the film covering the skin surface that accelerates the moisture dissipation from the fruit [38].

The mass loss of mangoes was influenced by the compound treatments and the storage time. The loss of moisture during storage is the main reason for the mass loss. In addition, the consumption of substances by the metabolic process of mango also decreases the mass of the fruit [30,39]. Zainal et al. also showed that the reduction in the fruit mass was associated with the softening of the peel and pulp [35]. The high mass loss of the CK and HF in the 12 days of storage indicate that the packaging had a limited effect on the protection of the mangoes during transportation, and that edible coating and MAP techniques had a greater impact on maintaining the freshness and quality of the mangoes. In contrast, the mass loss of the MAP-treated mangoes was significantly reduced throughout the whole storage, which is consistent with the results of the earlier studies [40]. That the MAP and low-temperature compound treatments could inhibit metabolic activities more effectively, and reduce the dissipation of moisture and nutrients, were the main reasons [41].

Mangoes continue to respire autonomously after harvest; however, respiration consumes substrates that cause irreversible changes in fruit nutrition, appearance, and flavor [11]. Respiration also causes tissue softening and relaxation, which reduces the storage characteristics of the fruit [42]. The higher the respiration rate of mangoes, the faster they ripen and age, affecting their postharvest longevity [36]. The edible coating compound treatment effectively inhibited the respiration rate during storage, and the results indicate that the fruit metabolism was inhibited and that the treatments improved the quality of the mangoes. The MAP compound treatment inhibited ethylene production and attenuated the stimulation of ethylene on fruit ripening [43]. The delay in the time of the peak respiration of the mangoes indicates that the MAP compound treatment delayed the aging process of the mangoes and had a better effect on the fruit preservation in terms of fruit appearance.

The cell membrane is a semipermeable membrane capable of the selective transport of substances [44]. Fruit cell membranes are subject to changes during storage by external and self-induced factors, and their selective properties can be compromised. The functional activity of the cell membrane can be reflected by the changes in the relative conductivity of the fruit, and the degree of injury and the resistance of the fruit to stress can be evaluated [45]. The relative conductivity of the CK was significantly higher than those of the other treatments in the prestorage period, indicating that the single packing treatment had limited protective effect on the fruit. The different treatments effectively suppressed the increase in the relative conductivity, mainly due to the low O_2_ and high CO_2_ microenvironment created by the edible coating or the MAP compound treatment for mangoes, which reduced or delayed the respiration rate of the fruit. At the same time, the compound treatments enhanced the antioxidant capacity and reduced the attack of free radicals on the cell membranes [46]. These effects resulted in the delay of the fruit senescence process and the inhibition of the increase in cell membrane permeability [47].

The TSS is an important index for evaluating the nutritional value and the storage characteristics of fruits containing sugars, organic acids, and minerals. The mangoes used in the experiment were initially immature, and the postripening effect caused the decomposition of organic matter in the mango fruit, which led to an increase in the TSS content [48]. Sugars are continuously consumed as substrates during respiration, which may cause the TSS to decline [49]. Controlling and maintaining the upward trend of the TSS is the goal of fruit storage. Compared to the CK, the different treatments inhibited the rise of the TSS in the mangoes during the storage, and the effect of the HM was more significant in the first 24 days. The inhibition of mango respiration by the MAP compound treatment slowed down the rate of fruit metabolism, which might be the reason for the lower TSS [50].

Firmness is an important indicator for evaluating the storability characteristics of fruits and is a quality attribute that affects consumer preferences. Fruit firmness usually decreases after harvesting because of postripening and the aging of the fruit. Pectin is a polysaccharide substance present in the cell wall and mesocosm layer of the fruit [51]. Protopectin is degraded by pectin methyl esterase, and other enzymes, at the postripening stage, which softens the fruit tissue and causes a decrease in firmness [52]. In addition, Zainal et al. reported a correlation between the reduction in moisture and mass and the decrease in fruit firmness [35]. The HPM and HM had a significant effect on the maintenance of the mangoes, such as the metabolism and moisture, so the firmness results were better than those of other treatments. The inflatable bag packaging method reduced the physical damage to the fruit, in comparison to the HPF and HF results.

The physiological metabolic activity of the fruit is still ongoing after harvest, and the oxygen radicals generated during this process attack the biofilms and trigger lipid peroxidation [53]. Some lipid peroxides cause cellular damage, affect metabolic processes, and even trigger cell death [54]. Therefore, malondialdehyde, as a type of lipid peroxide, can reflect the degree of lipid peroxidation in the organism and evaluate the damage suffered by cells. The compound treatments inhibited the increase in the MDA content of the mangoes; however, the HF was not effective after 18 days of storage. The HF treatment showed more spots and a rapid decrease in the fruit quality at the end of storage, which may be an important reason for the rapid increase in the MDA content [55]. The HPF aerated in the inflatable bag was significantly lower than the HF, indicating that this packaging method reduced the transport damage to the fruit and improved the storability. The MAP compound treatment inhibited the metabolic activity of the fruit and improved the oxidative capacity of the organism (combined with antioxidant enzyme analysis). Therefore, the increase in the MDA of the HPM was significantly lower than that of the CK throughout the storage.

Titratable acid is the main factor affecting the flavor of the fruit, and medium acidity with high sugar content is a characteristic of good quality and intense flavor [56]. Titratable acids contain a variety of organic acids, which are substrates for the respiratory metabolism of the fruit [52]. Therefore, the TA content decreased continuously with increasing storage time. The TA content of the CK decreased rapidly during the first 6 days of storage; however, the treatments significantly suppressed this trend and continued until the end of storage. This is mainly due to the fact that the compound treatments reduced or delayed the respiration rate and slowed down the process of maturation and senescence [15]. The TA content of the HPF was 2.22 and 1.69 times that of the CK on the 6th and 12th days, respectively, but the HPM and HM retained more of the TA content at the end of storage. The rapid increase in the edible coating compound treatment spots made the fruit quality decline rapidly at the end of storage, which was similar to the results of the disease index and the MDA content. The MAP compound treatment retained a good appearance at the end of storage, and the delayed respiratory peak may allow the fruit to consume less total substrate and maintain a higher TA content [43].

Vitamin C is a necessary nutrient for humans and is mainly found in reduced form (L-ascorbic acid) in fruits; thus, it has a highly effective antioxidant effect [57]. The VC can scavenge oxygen free radicals, reduce cell damage, and slow down fruit aging [58]. At the same time, VC is easily oxidized during storage, and its content generally tends to decline. The edible coating on the surface of mangoes could effectively reduce the exposure to oxygen and decrease the oxidative enzyme activity in the organism, thus delaying the decline of VC content [47]. The MAP treatment, on the other hand, directly reduced the oxygen in the mango storage environment, thus decreasing the oxidative loss of VC. Shi et al. reported that acidic conditions could increase the stability of VC [59]. The HF and HPF maintained more VC content during storage but were lower than the MAP compound treatment on the 30th day. The MAP aerobic environment may have resulted in more VC loss than the edible coating compound treatment in the prestorage period, but the inhibition of fruit metabolism by the MAP compound treatment resulted in less total VC loss.

The SOD and POD can scavenge superoxide anion radicals generated during the physiological activities of mango, thus reducing the oxidative effects of free radicals on tissue cells [60]. In addition, POD is also beneficial to maintaining the balance of hydrogen peroxide in the cell wall and enhancing the defense ability of the fruit against external adverse factors [61]. Therefore, these two antioxidant enzymes can slow down the process of fruit peroxidation and maintain the integrity of the membrane structure and postharvest quality [62]. The higher contents of SOD and POD indicate that the compound treatments improved the ability of mangoes to scavenge oxygen radicals and reduced the damage of free radicals on the macromolecules [10]. The edible coating, or MAP compound treatment, inhibited physiological and biochemical reactions, and delayed fruit senescence, which was why the fruits still had a strong antioxidant capacity in late storage [63]. However, more studies of the defense enzyme systems are needed in order to determine the differences that cause the different treatments to behave differently on the SOD and POD contents.

## 4. Materials and Methods

### 4.1. Materials and Treatment

Mangoes were sourced from Shanghai Daozhi Agricultural Products Co., Ltd., Shanghai, China. The fruits were selected as test materials with uniform maturity, no disease spots, and no physical damage. The mangoes were wiped of surface dirt, the pedicles were trimmed, and they were cleaned of secretions and subsequently prechilled for 12 h at 13 °C. Then, these mangoes were randomly divided into 5 groups, each with 30 mangoes, and they were treated with different compound preservation treatments (Table 2).

The polyethylene foam mesh sleeve was a commercial packaging method with limited effect on mango protection, and an inflatable bag was a better vibration mitigation packaging method in our preliminary study. The formulation of the edible coating was 2.5% chitosan (200–400 mPa.s, Shanghai Aladdin Biochemical Technology Co. Ltd., Shanghai, China), 0.3% zein from corn (Tokyo Chemical Industry Co. Ltd., Chuo-ku, Tokyo, Japan), and 0.2 g kg^−1^ natamycin (Shanghai Aladdin Biochemical Technology Co. Ltd., Shanghai, China). A simulated transport shaker (LX-100VTR, ASTM compliant, Shanghai Luxuan Instrument and Equipment Factory, Shanghai, China) was used for the 24-h transportation treatment, which was set at 180 r min^−1^ of rotation speed, and 3 Hz of vibration frequency in the vertical direction. The better treatment conditions in our previous study, 7% CO_2_ + 3% O_2_ + 90% N_2_, were used for the MAP conditioning (JY-500, Shanghai Jiyi Machinery Co., Ltd., Shanghai, China). All mangoes were treated according to Table 2, and then stored in a cold room at 13 °C. The start of storage was recorded as Day 0 and, subsequently, samples of 6 fruits at a time were measured every 6 days.

### 4.2. Fruit Color and Disease Index

The packaging and edible coating were removed and dried before scoring by a professional scoring team. The yellowing degrees and disease indexes of the mangoes were obtained by scoring the percentage of the yellowing and disease spots on the peel, and the evaluation criteria were performed according to Xiao et al. [53] and Esquivel-Chávez et al. [64], respectively. The pulp of each treatment of mangoes was taken out homogenized, and then the pulp color characteristics were measured using a colorimeter (CR-400, Konica Minolta Co., Ltd., Tokyo, Japan). The total color difference (Δ*E*) was calculated according to Equation (1) [65].

(1) $$\Delta E=\sqrt{{\left(\Delta a\right)}^{2}+{\left(\Delta b\right)}^{2}+{\left(\Delta L\right)}^{2}}$$

### 4.3. Physicochemical Analysis

The physicochemical properties, including the moisture distribution, mass loss, respiration rate, relative conductivity, and total soluble solids (TSS) of the mangoes were determined during the storage. Three mangoes were randomly selected from each treatment, and a pulp piece of approximately 5 cm × 3 cm × 1 cm was taken at the equator. The pulp moisture distribution was examined in a low-field NMR analyzer (MesoMR23-060H-I, Suzhou Niumag Analytical Instrument Corporation, Suzhou, Jiangsu, China) according to the description by Kirtil et al. [66]. The transverse relaxation time, T2, was determined using the CPMG sequence. The weight of the mangoes was measured at the initial and each evaluation period, and calculated as a percentage of the mass loss to the initial weight [67]. The respiration rate was measured according to Sheng’s method and the results were expressed as CO_2_ mg kg^−1^ h^−1^ [68]. Before the relative conductivity measurement, a total of 30 discs, with a diameter of 5 mm and a thickness of 2 mm, were removed from each treatment of mangoes. To determine the relative conductivity of the mangoes during storage, the values after 30 min of shaking were calculated (conductivity meter, Mettler-Toledo Instruments (Shanghai) Co., Ltd., Shanghai, China) as a percentage of that after 30 min of a boiling water bath [69]. The TSS content was determined using a portable refractometer (LB30T, Guangzhou Mingrui Electronic Technology Co., Ltd., Guangdong, China) and the results were expressed as Brix%.

### 4.4. Quality Analysis

Mango fruits from different treatments were evaluated for the firmness, titratable acid (TA), vitamin C, and malondialdehyde (MDA) contents. To determine the firmness of the mangoes, six loci were measured for each mango using the texture analyzer (TA-XT Plus C, Stable Micro Systems Co., Ltd., Surrey, United Kingdom) SMS-P/2 cylinder [23]. The mean values of all the measurement points in each treatment were calculated and the results were expressed in g. The MDA content in the mango fruit pulp was determined by kit (Nanjing Jiancheng Institute of Bioengineering, Nanjing, China), and the results were expressed as nmol g^−1^. The TA content was determined using acid–base titration and the results were expressed as a percentage of the citric acid [58]. The vitamin C content in the mango pulp was determined using 2,6-dichloroindophenol, according to the method of Chen et al. [70]. The results were expressed as g 100 g^−1^ fresh weight (FW).

### 4.5. Antioxidant-Related Enzyme Analysis

The superoxide dismutase (SOD) and peroxidase (POD) contents in the mango pulp were determined using POD and SOD enzyme kits (Nanjing Jiancheng Institute of Bioengineering, Nanjing, China), respectively, according to the instructions. The results are all expressed as U g^−1^ FW.

### 4.6. Statistical Analysis

All data were repeated at least three times, except for special instructions, and the means ± standard deviation were taken. Analysis of variance (ANOVA) was performed using IBM SPSS Statistics 25.0 (Version 25.0, IBM SPSS Statistics, New York, NY, USA), and significance was performed using Duncan’s multiple range tests, with significant differences when *p* < 0.05. Data statistics were performed by Microsoft Excel 2019 (Microsoft Corporation, Redmond, WA, USA), and figure legends were drawn using Origin 2019b (Version 2019b, OriginLab Corporation, Northampton, MA, USA).

## 5. Conclusions

The compound treatments retained more free moisture, TA, and VC content of the mangoes, and maintained the fruit firmness and superior quality characteristics. Antioxidant enzymes enhanced the fruit resilience, reduced cell damage, and inhibited the increase in the MDA, relative conductivity, and TSS. The compound treatments also significantly reduced the yellowing degree, disease index, and mass loss. The use of inflatable bag packaging for transportation reduced the physical damage to the fruit, and the interaction of the edible coating, MAP, and low-temperature treatments reduced the respiration rate and slowed down the aging process. Fruits with the compound preservation treatments, especially the HPM, had significantly better overall visual acceptability and quality than the CK. Therefore, the HPM compound treatments can be used to extend the shelf life and commercial characteristics of mango fruits. The edible coating compound treatment can also be used as a green means of fruit preservation, but further enhancement of fruit disease control is needed.

## Figures and Tables

**Table 1:** Color characteristics of mango pulp with different treatments, stored at 13 °C, on the 24th day.

Treatments	L*	a*	b*	Δ*E*
CK	46.4 ± 0.78 ^c^	−0.36 ± 0.20 ^a^	37.5 ± 1.09 ^d^	59.8 ± 0.59 ^b^
HF	46.8 ± 0.51 ^c^	−1.83 ± 0.25 ^c^	40.8 ± 1.41 ^a,b^	61.5 ± 1.14 ^a^
HM	50.8 ± 0.89 ^a^	−0.73 ± 0.05 ^b^	38.9 ± 0.89 ^c,d^	57.2 ± 0.66 ^c^
HPF	48.7 ± 0.75 ^b^	−1.71 ± 0.32 ^c^	39.4 ± 0.46 ^b,c^	59.2 ± 0.71 ^b^
HPM	51.5 ± 0.56 ^a^	−0.68 ± 0.13 ^b^	42.0 ± 1.61 ^a^	58.7 ± 1.08 ^b^

Different letters indicate significant differences between treatments for the same storage time by Duncan’s multiple range tests (*p* < 0.05). ΔE indicates a total color difference. Treatments: CK (Control): Inflatable bag packaging + Vibration; HF: Coating + Foam mesh sleeve packaging + Vibration; HM: Foam mesh sleeve packaging + Vibration + MAP; HPF: Coating + Inflatable bag packaging + Vibration; and HPM: Inflatable bag packaging + Vibration + MAP.

**Table 2:** Different compound preservation treatments.

Groups	Treatments
CK	Inflatable bag packaging + Vibration + Cold Storage
HF	Coating + Foam mesh sleeve packaging + Vibration + Cold Storage
HM	Foam mesh sleeve packaging + Vibration + MAP + Cold Storage
HPF	Coating + Inflatable bag packaging + Vibration + Cold Storage
HPM	Inflatable bag packaging + Vibration + MAP + Cold Storage

## Data Availability

Not applicable.
